# Approaches to Exceptional Points in the Framework of Non-Hermitian Random Matrices

**DOI:** 10.3390/e28020149

**Published:** 2026-01-29

**Authors:** Henri Benisty

**Affiliations:** 1 Laboratoire Charles Fabry, IOGS, Université Paris-Saclay, 2 Av. Fresnel, 91120 Palaiseau, France; henri.benisty@institutoptique.fr; Tel.: +33-1-6453-3286; 2 LIED Laboratory, Université Paris Cité, 75013 Paris, France

**Keywords:** random matrix theory, Petermann factor, overlaps, exceptional points, eigenvalue, perturbation, Ginibre ensemble

## Abstract

We explore how easy it is to enforce the advent of exceptional points starting from random matrices of non-Hermitian nature. We use the Petermann factor, whose mathematical version is called “overlap”, for guidance, as well as simple pseudo-spectral tools. We attempt to proceed in the most agnostic way, by adding random perturbation and checking basic metrics such as the sum of all vectors’ Petermann factors, equivalently the sum of diagonal overlaps. Issues such as the location of high Petermann factors vs. the modulus of eigenvalue are addressed. We contrast the fate of exploratory approaches in the Ginibre set (real matrices) and complex matrices, noting the special role of exceptional points on the real axis for the Ginibre matrices, completely absent in complex matrices.

## 1. Introduction

The role of non-Hermitian (NH) operators is crucial in several branches of physics. In classical physics, coupling of a large number of damped oscillators can naturally make use of such NH matrices, with the eigenvalues characterizing the eigenmodes’ oscillatory frequencies in the real part and damping in the imaginary part [1,2]. In linear physics, the desire to tame the imaginary part of the eigenvalues thanks to various flavors of coupling engineering has been examined under several respects: in biological neural networks in works such as [3], for instance, or in the physics of so-called quasi-normal modes in nanophotonic resonators [4,5,6,7] whose radiation and Joule losses are introducing specific categories of damping [8,9,10]. In acoustics, musical-anthropological studies of 20th-century instruments, such as steelband drums, hinge on NH physics, with the choice of up to 24 “notes” cleverly distributed onto the drum’s active area [11,12].

A singular feature attained in some NH operators is the exceptional point (EP), whereby the eigenvalues and eigenvectors are merging. It has attracted a lot of attention in the context of parity-time symmetry (PTS), initially an abstract concept, which appeared, however, to be a staple of modern photonics since 2007, as the gain and loss parameters of photonic materials give rise to configurations that satisfy, in essence, the PTS requirements or closely related ones [2,8,9,13,14,15]. Among several issues, the possibility to display enhanced sensitivity to perturbation in a physically meaningful way has been explored, and various controversies have been tackled [16,17,18,19,20,21] involving the physics of higher-order EPs whereby three or more eigenvalues merge. The Petermann factor that we introduce below is a measure of the perturbative sensitivity of the eigenstate to the NH matrix entries.

Note that the dimension of EPs is an important aspect. Here we deal with “EP2”, where two eigenvalues and eigenvectors coincide, but higher-order EPs, so-called “EP*n*” (*n* > 2), are also possible. On the other hand, depending on the size of the parametric space, the obtainment of higher-order EP and of further singularities such as arcs (e.g., for loci where only real parts merge) is constrained by the space co-dimension 2*n* − 2 and by symmetries [22]. As we consider different entries of the random matrices in this paper, we implicitly deal with different situations in this respect, as we will comment in the bulk of the study.

For completeness, it is also worth mentioning that Hopf bifurcation in nonlinear physics is described also by an EP of the linearized Jacobian matrix in the bifurcation area [23,24]. Indeed, nonlinear behavior of coupled oscillators, for instance, can mimic gain or loss (think of “parametric gain” in photonics and similar phenomena in acoustics), which makes the analogy not completely surprising.

In general, the physics of EP is also interesting because of the possible trajectories followed by a system when it encircles such EPs, with the multiple Riemann sheets of eigenvalues. Any nonlinear phenomena (saturation, noise overcoming vanishing components) can then lead to “jumps” among sheets, thus triggering a series of interesting behaviors.

One simple feature observed early in PTS systems is the fact that “near-EPs” often characterize real systems due to complex coupling; for instance [25,26], a shift from an ideal EP, which appears easy to correct, for inst by a small detuning of the interacting oscillators. However, for larger dimensional systems, there is no clear way to perform such a “correction”, i.e., to force the appearance of EPs through small changes to the original NH operator. This questioning brings us into the realm of (large) random matrices and RMT (random matrix theory), obviously with NH matrices, whose two main categories are the Ginibre ensemble (real coefficient, non-symmetric) and the general complex random matrices. While EPs are simple to visualize for small matrices (up to 4 × 4, say), tools more adapted to the “agnostic” situation of RMT are advantageous to tackle the issue that we want to address in this paper: “how can a given NH operator be modified by small amounts to exhibit at least one EP, and in what part of the spectral domain (complex plane) should we expect this EP to lay.” The most adequate tool to tackle this issue is the “overlap” (in mathematics) or the so-called Petermann factors. To the best of our knowledge, even though the topic received interesting contributions in the physics of quantum chaos [27], it is the mathematical community that has mostly explored this area, in several papers spread over two decades, such as [28,29,30,31,32,33,34].

Mathematically, since the right and left eigenvectors differ in NH matrices [18,28,34], the so-called “overlaps” are what measure this difference [28,29,32]. The diagonal overlaps are simply $\langle R_{i}^{*}R_{i}\rangle \langle L_{i}^{*}L_{i}\rangle$ where $R_{i}$ and $L_{i}$ are the right and left eigenvectors, and the product is the conventional inner product of Hilbert space [17,18]. Diagonal overlaps diverge when approaching EPs, under the convention that biorthogonality is written as $L_{i}^{t}R_{j}=\delta_{ij}$. The abovementioned Petermann factors (PF) describe essentially the same thing but divided by ${\left|\langle L_{i}^{*}R_{i}\rangle \right|}^{2}$. Due to the Cauchy–Schwarz inequality, PF are clearly larger than 1, and their definition does not rely on the biorthogonality convention. PF calculation is straightforward when the vectors are normalized, i.e., $\langle R_{i}^{*}R_{i}\rangle =1$, as performed in the matlab linear algebra package used in this work: ${PF=\left|\langle L_{i}^{*}R_{i}\rangle \right|}^{-2}$.

The first goal of our paper is to explore more visually than through the rendition of mathematics papers the distribution of PF in random NH matrices, either Ginibre ensemble or fully complex. The next goal is to answer the issue raised above: What happens upon exploring “agnostically” the evolution of random matrices? Can we add small amounts of random matrices to a given one that eventually lead to the emergence of one EP (generally absent in the initial matrix)? We simply perform an algorithmic study in this respect and show that “weak” efforts can suffice to lead to the emergence of such EPs. We check the properties of the resulting operator by using simple pseudo-spectral analysis [35]. The paper follows the above program: in the next Section 2, we illustrate the distribution of overlaps in sets of random matrices for relatively small matrices (where the circle law is still substantially violated). We notably point out the scarcity of large PF at large eigenvalues, already notable in the mathematical works. In Section 3, we show the result of modifications of the initial matrix. For the general complex matrix, we proceed by small steps. For the Ginibre matrix, we show that the path connecting two independent matrices (i.e., their “mix” in variable proportions) naturally gives rise to several EPs when the eigenvalues merge on the real axis, which a fraction of them are bound to cross at some point. In Section 4, we discuss the results and in Section 5 we conclude.

## 2. Visualizations of PF Distribution in Ginibre and Complex Matrices

We extracted the eigenvalues and right as well as left eigenvectors of a large number of N×N random matrices with i.i.d. entries having either the form $\frac{N\left(1\right)}{\sqrt{N}}$ for Ginibre matrices (asymptotic spectral radius = 1) or $\frac{N\left(1\right)}{\sqrt{N}}+i\frac{N^{\prime }\left(1\right)}{\sqrt{N}}$ for complex matrices, with $N\left(1\right)$ and $N^{\prime }\left(1\right)$ the normal Gaussian distribution. We operate at N=24 and about $≲10^{6}$ matrices to frugally handle numerics. At such low values, the semicircle distribution of eigenvalues (radius 1 for real matrices or $\sqrt{2}$ for complex ones) has substantial tails, but as one of the issues is the exploration of EP at large eigenvalues, this makes the study easier.

**Figure 1:**
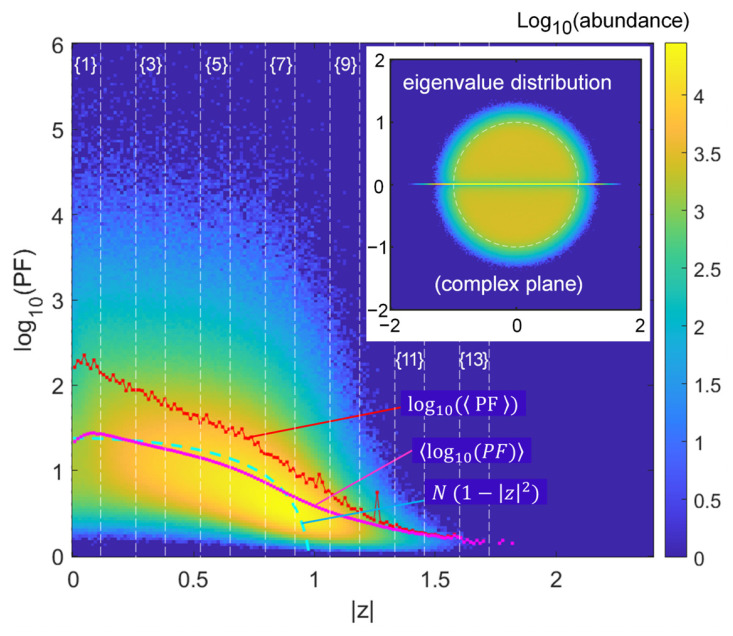
Ginibre matrices. Distribution of the PF vs. eigenvalue modulus $\left|z\right|$ for N=24. The ordinate is $\log_{10}\left(PF\right)$ and the histogram abundance is itself rendered through its log. Lines describe the $N(1-{\left|z\right|}^{2})$ law (cyan), the mean of the apparent cloud of abundance ($\langle \log_{10}\left(PF\right)\rangle$ magenta) and the mean of the PF ($\log_{10}\left(\langle PF\rangle \right)$, red), lying well above the former as is logical with a tail of very large PFs causing the spiky result, most apparently at $\left|z\right|≃1.25$. The inset shows the eigenvalue distribution with the real axis cluster. The thin vertical lines delineate the clusters in Figure 2 to study the tails.

We first present the results as a 2D histogram of the Ginibre case in the $\left\{\left|z\right|,\log_{10}\left(PF\right)\right\}$ plane binned over 180 bins in Figure 1, with abundance itself in log scale to observe the tails distribution in sufficient detail (note that in subsequent Figure 5 a few contours at log-scaled abundances of the histogram help capture the represented trends). There is a small dip in abundance around $\left|z\right|≲0.1$, that can be attributed to the crowding of eigenvalues on the real axis, where they are repelled from each other and cannot populate the origin area around $\left|z\right|=0$ as much as the rest.

The spread is thus very large around the trend of the $N(1-{\left|z\right|}^{2})$ Chalker–Mehlig law [28,29]. The plot of the $\langle \log_{10}\left(PF\right)\rangle$ added on the graph only exhibits a section around $\left|z\right|\sim 0.5$ to $\left|z\right|\sim 0.75\;$ where this trend is somehow reproduced, but extremes deviate substantially. The trend of mean PF itself (thus $\log_{10}\left(\langle PF\rangle \right)$, plotted in the log scale as well) is not similar, being “upward-biased” by the not-so-few large extreme values of PF. It is also “spiky”, due to the random nature of these extremes dominating the average. The central $\left|z\right|$ range with the highest abundances of large PFs might even cut-off a couple of spikes due to the histogram truncation at $\log_{10}\left(PF\right)=6.$

**Figure 2:**
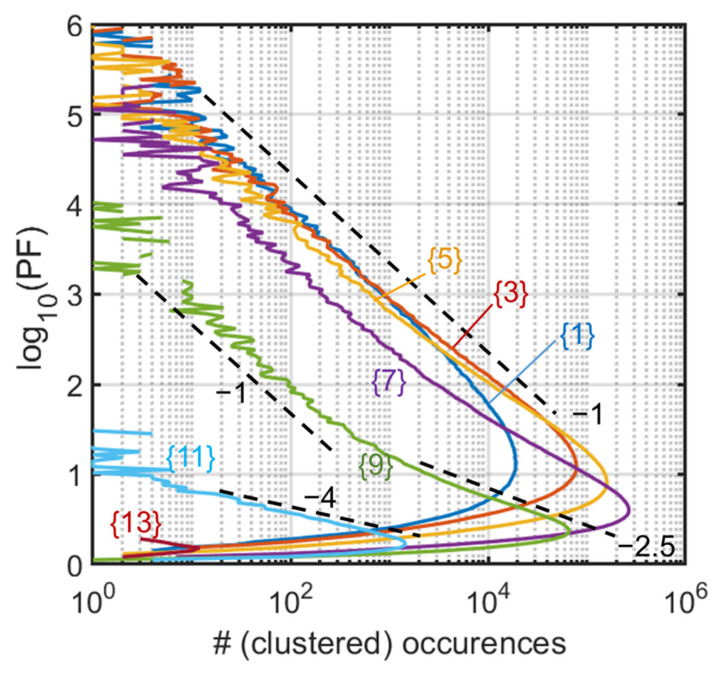
Ginibre matrices. Histograms along stripes of width $\Delta \left|z\right|=$ 0.133 and start positions $\Delta \left|z\right|\times \{1,3,5,7,9,13\}$ as indicated (colors). Slopes of the tails are −1 (dashed lines) for the lower start positions ($\left|z\right|<1$ up to {7}), but include a more and more steep section (dashed lines of slope −2.5 and −4) after the peak for the larger ($\left|z\right|>1$) cases {9} and {11}.

To further characterize the trend of the tail, we cluster the bins of $\left|z\right|$ in nine bigger bins of width $\Delta \left|z\right|=$ 0.133 and start positions $\left|z\right|=\Delta \left|z\right|\times \{1,3,5,7,9,13\}$, as indicated in corresponding vertical stripes in Figure 1 (labeled {1} to {13}). In Figure 2, we can thus analyze the trend of the high-PF tail, associated mainly to the −1 slope. Larger slopes such as −2.5 and −4 are seen for the larger $\left|z\right|$ values in limited ranges of log, but in a region of scarcer abundance anyway (beyond the unit circle). Note that the −1 slope means here an abundance scaling as $PF^{-1}$, at the limit of a fat tail. We will elucidate the role of the central line of the distribution of eigenvalues (the central line on the real axis of the inset of Figure 1) by comparing this result with that of complex matrices. We notably suspect that the change in slope visible in case {9} is highly related to the possibility for high PF values to be produced close to the real axis, but only if density of eigenvalues is sufficient, hence not at the largest $\left|z\right|$ values. A kind of cross-over behavior could thus appear and manifest itself by the distinct slopes, with some points samples far from the real axis, and others closer contributing in proportions that differ much from the other slices in this intermediate case. We will also see that the steeper slope translates in a difficulty to raise the Petermann factor when it concerns large eigenvalues, an important issue since those large eigenvalues tend to dominate the response in some way, in numerous physical implementations or models using NH matrices.

To conclude this first study, we found that, not so surprisingly, the Ginibre set, in spite of its quicker calculation, displays numerous complexities.

**Figure 3:**
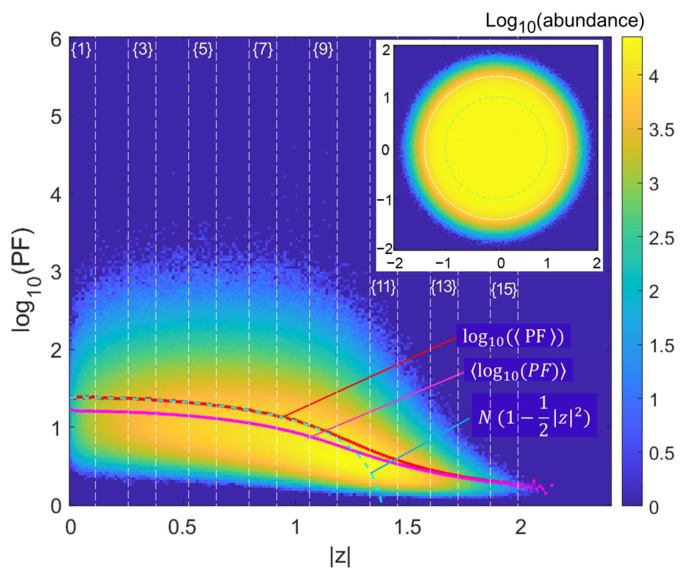
Complex matrices. Distribution of the PF vs. eigenvalue modulus $\left|z\right|$ for N=24. The ordinate is $\log_{10}\left(PF\right)$ and the histogram abundance is itself rendered through its log. Lines describe the $N(1-\frac{{\left|z\right|}^{2}}{2})$ law (cyan), the mean of the apparent cloud of abundance ($\langle \log_{10}\left(PF\right)\rangle$ magenta) and the mean of the PF $(\log_{10}\left(\langle PF\rangle \right)$, red), now coinciding with the expected law in all the range $\left|z\right|\leq 1.1$. The inset shows the eigenvalue distribution and the asymptotic radius $\sqrt{2}$ (white dotted) as well as the unit circle (cyan, dashed).

We now study complex matrices of the same size and with the same numerical sampling to review the histogram of PF. As we use the entries $\frac{N\left(1\right)}{\sqrt{N}}+\frac{N^{\prime }\left(1\right)}{\sqrt{N}}$, the reference (asymptotic N→+∞) spectral radius is $\sqrt{2}$. We therefore adapt the Chalker–Mehlig law as $N(1-\frac{1}{2}{\left|z\right|}^{2})$. In Figure 3, we thus represent the the same kind of histogram that was performed for Ginibre matrices, with same parameters ($10^{6}$ draws of N=24 matrices).

Obviously, the distribution is now much gentler with much less very large extreme values of PF. Only scarce PF occurrences exceed $10^{4}$. Accordingly, the ratio between the PF mean $\log_{10}\left(\langle PF\rangle \right)$ and the log-mean, $\langle \log_{10}\left(PF\right)\rangle$ (the cloud apparent mean) is now about 1.16 at lower $\left|z\right|$ values (peaking at 1.18 for $\left|z\right|≃1$, and trending to 1 at larger $\left|z\right|≃2$). There is now a very good agreement with the $N\left(1-\frac{1}{2}{\left|z\right|}^{2}\right)$ law, up to $\left|z\right|=1.1$, i.e., when the asymptotic circle radius $\sqrt{2}$ is approached within $\sqrt{\frac{2}{N}}\sim \;0.3$, the typical scaling of extreme eigenvalue tail effects adapted to our context.

**Figure 4:**
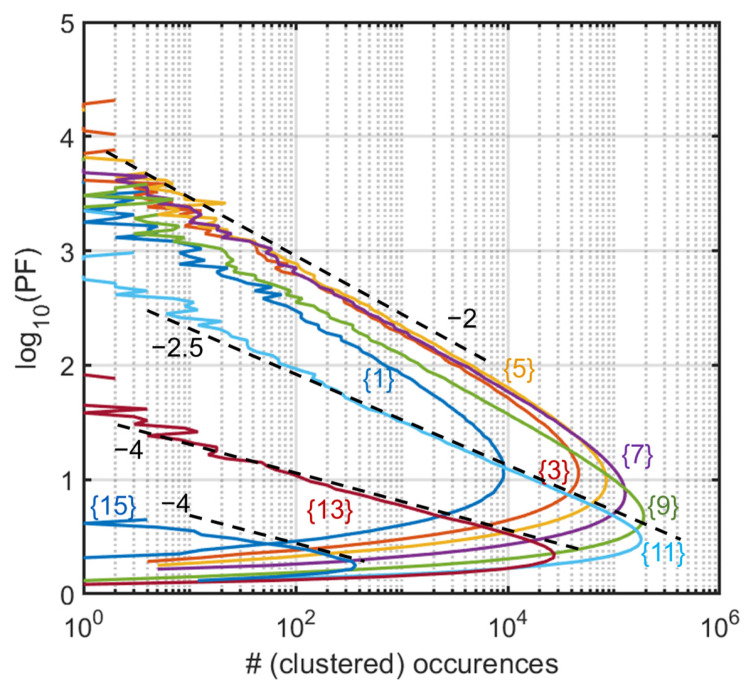
Complex matrices. Histograms along stripes of width $\Delta \left|z\right|=$ 0.133 and start positions $\Delta \left|z\right|\times \{1,3,5,7,9,13,15\}$ as indicated (colors). Slopes of the tails are −2 (dashed lines) for the lower start positions ($\left|z\right|<1.2$ up to {9}), but their decay becomes wholly steeper for the larger ($\left|z\right|>1.2$) cases, with {11} and {13} being around a −4 trend and possibly steeper for the last slice {15}.

Performing the same “stripe analysis” as for the Ginibre matrices, we now find a much faster decay, with a −2 exponent for the main stripes (stripes {1} to {9}) (Figure 4).

The stripes are the same as above, so the stripe number is not scaled with the asymptotic radius ($\sqrt{2}$ vs. 1). Asymptotic slopes for $\left|z\right|<1.2$ (stripes {1} to {9}) are now clearly around −2 instead of −1 previously. As we move to the rightmost stripes ($\left|z\right|>1.2$, stripes {11}, {13}, and {15}, now populated), the slope increases steadily (for abundances covering typically three decades), although the largest strip is too noisy to provide a bona fide slope.

Note for completeness that studies at much larger N~400 have also been performed, albeit with reduced samples. It provided similar results except for the narrower intermediate region at the spectral radius limit, due to its well-known scaling as $1/\sqrt{N}$.

To conclude this section, we see that the issue of extreme PFs (or overlaps) is largely affected by the Ginibre vs. complex matrix eigenvalue distribution, with the specificities of the Ginibre matrices causing a large tail scaling essentially as $PF^{-1}$, vs. $PF^{-2}$ for complex matrices, and deviations from the Chalker–Mehlig prediction that are substantial, notwithstanding the specificities of the well-studied spectral radius area asymptotics. As for the transition between the two regimes, it appears to be caused by an imaginary part of the order of 2 × 10^−3^ vs. the real part: We probed that a choice of entries such as $\frac{N\left(1\right)}{\sqrt{N}}+i\frac{N^{\prime }\left(1\right)}{4N^{2}}$ (thus for our case $\left(\frac{1}{4}N^{-1.5}\right)\sim 0.0022$) led to typical intermediate tail behaviors with slopes $\sim PF^{-1.5}$ but with less clear-cut behaviors than in limit cases.

Note also that the likeliness of large PFs is directly correlated with the spacing statistics of eigenvalues. It is well known that in the complex NH case, the spacing of nearest eigenvalues (with the metric $s=|\lambda_{i}-\lambda_{j}|$) follows a cubic law $P\left(s\right)\propto s^{3}$ near s=0, which means a substantial degree of repelling. Conversely, in the real NH (Ginibre) case, one observes $P\left(s\right)\propto s$, but a simple analysis shows that this contribution stems from the eigenvalues with $\left|Im(\lambda )\right|≲N^{-1/2}$, i.e., the equator, not from the rest of the disc where $P\left(s\right)\propto s^{3}$ is found. This also means that the PF may safely be used to quantify the distance to the nearest neighbor eigenvalues without the need to calculate the whole spectrum or even the local spectrum. This repelling behavior in most of the disc is also a justification of the need for a strategy to seed EPs as proposed in the next section.

While these results are probably a part of existing mathematical knowledge, there was, to the best of my knowledge, no visual rendering of the empirical trends associated with the mathematical formulations on these sets of eigenvectors. In order to tackle the issue of “restoring” or “creating” or “seeding” EPs, thus diverging PF, it can be useful for a large part of the applied physics audience (acoustics, photonics, electronics, other engineering subfields…) to grasp the actual trends through such tools in order to get some agency on the advent (or avoidance) of EPs in the various domains concerned by them, in linear and nonlinear physics.

## 3. Pushing Non-Hermitian Matrices Towards EPs

We now consider the issue of generating EPs in a given starting system. In more algorithmic terms, we apply a series of additive corrections to a given matrix M that generally has limited PF typically less than ~$10^{2}$ as can be inferred from the above stripe analysis: there are ~2 eigenvalues per stripe, distinct or not depending on the case, and the part above ~$10^{2}$ is at most a few percent for the fattest-tailed stripe. For each additive correction, we check whether the largest PF has increased. We can also target this estimator by bracketing it to those PF associated with the largest eigenvalues, obviously scarcer cases, to raise the difficulty of finding a hit. The size of the correction, of course, matters: as one approaches an EP, vanishing steps must be performed to close in and avoid excess corrections. We do not try in this first investigation to get into algorithmic optimization of the process; we do not doubt that it would reveal interesting mathematics and would find applications possibly in those areas where EPs are regarded as an interesting working point for actual physical systems. But we mainly want to check that the simple ideas of the elementary cases of “EP healing” that were developed in the previous decade [25,26] have a correspondence in the context of random matrices, sticking to the “agnostic” concepts that underlie the use of RMT and taking their universal character as a possible benefit.

**Figure 5:**
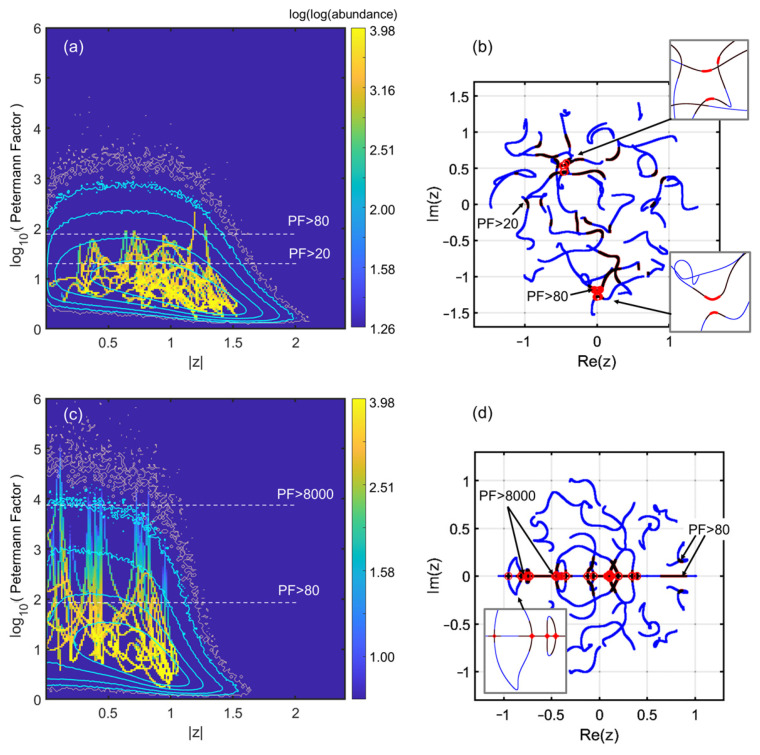
Path scan $M\to M^{\prime }$ between two random matrices using 400,000 points. (**a**,**b**): complex matrices; (**c**,**d**) real (Ginibre) matrices. The eigenvalue paths are shown in (**b**,**d**) in the complex plane. Loci of the higher PF values are outlined. Dark red (PF>20) and bright red circles (PF>80) for (**b**), complex matrices, and the same colors but thresholds PF>80 and PF>8000 for (**d**), real matrices. The PF are shown in the same spirit as the histograms (with a log(log)-scale color map for abundance), forming the coiled patterns. The histograms in cyan are those stemming from Figure 1, Figure 2 and Figure 3, with cyan contours drawn at abundances 5000, 1000, 100, and 10 from inside to outside, and the last fuzziest contour for unit abundance in light brown. In (**b**,**d**), the square insets show typical large PF areas, showing that they do not occur on the most “wiggly” features in case (**b**), and that they resemble the familiar patterns of parity-time symmetry eigenvalue trajectories [25,26] in case (**d**).

A heuristic step to get a clue on the landscape leading to high PF is to simply inspect what happens upon scanning between two given NH matrices, i.e., a path $M\to M^{\prime }$ in matrix space, obtained by linear combination and normalization. It is a convenient way to be agnostic on how much and where high PF would be found. We present the result of such an investigation in both cases of real (Ginibre) matrices and complex matrices. We simply do a fine scan (4 × 10^5^ points) and present the result with the same kind of histogram previously used. The single matrix-to-matrix scan results in a pattern of curves sampling the various PF peaks, while we use contours of the maps of Figure 1 and Figure 3 as guides that help locate the loci of large PF. The two samples for the real (Ginibre) and complex cases provide the content of Figure 5a–d.

In Figure 5a we show the “histogramized” scan for the complex case, and the eigenvalue trajectories in Figure 5b. We outline paths featuring PF>20 in dark red and paths featuring PF>80 by light brown circles, associated to the limiting white dashed lines in Figure 5a. We notice that a few peaks emerge as our trajectories “scan” the normalized path between the two matrices. The peak values follow the trend of Figure 1, as the contours correspond to abundances of 10, 100, 1000, and 5000 from the outside to the inside (a fuzzier contour at abundance 1 was also added in light brown). Note that the fact that the motion can be “tracked by the eye” (i.e., there are prominent lines throughout the histogram) indicates that few singularities are approached. In other words, there is essentially a smooth evolution skirting near a few singularities. Thanks to Figure 5b we see that trajectories at the highest PF loci are not that wiggly. Patterns are of the anticrossing types, but with larger separation and more modest singularity than many of the smaller wiggles that do not give rise to high PF. Note also that along each trajectory the overall statistics (cyan contours) impact the pattern through the fact that the few uprising lines tend to be bent toward the favorable area around $\left|z\right|≲1$, where contours peak up.

In the case of the complex matrix, there are no large PF peaks. Conversely, for the Ginibre case, we do have a few genuine singularities, where the PF diverges. These can be easily tracked to eigenvalues crossing the real axis. Upon each such arrival (or departure) at the real axis, there is indeed an EP. This is relatively obvious, as the production of an eigenvalue pair cannot proceed differently than by an EP-type merge on the real axis before departure to the complex plane (or vice versa, on arrival).

This exploration therefore reveals that algorithmic ways to attain large PF could be very different in the two cases investigated. In the Ginibre case, the natural choice is to look for large PFs on the real axis, which entails possibly different strategies from perturbative ones. For the complex case, we are closer to the general idea that has inspired the present paper: there should be an EP “nearby” in the parameter space, accessible in a near-perturbation regime. In both cases, the EP scarcity at large $\left|z\right|$ means that the quest shall face sharply increasing difficulties as we get on the right of the histograms of Figure 1, Figure 2 and Figure 3 (or their cyan equivalent of Figure 5a,c).

We now consider how to attain an EP in a perturbative spirit. Since any path between two random matrices shall cut through several EPs in the case of real (Ginibre) matrices, the quest just amounts to locating a functional zero or pole, which can be made with known techniques (notwithstanding possible improvements due to the specific context of NH operators). Thus there is more interest to the complex case, at least heuristically, in order to parallel the quests of “healing EPs” made on small model systems, for which the equivalent of the “detuning” strategy for a 2 × 2 matrix is not trivial.

The idea, algorithmically speaking, is to make a perturbation with a random matrix again, and to select the perturbed version as the new one if its PF maximum, max(PF), is larger than the initial one. The strength of the perturbation, however, should be diminished as we approach an EP, otherwise we are scanning endlessly through the “cloud” that is commonly drawn in pseudospectral methods (that we shall address also later), and the convergence would be as fast as a random draw in this cloud can allow. So, we diminish the strength of the perturbation in the form B/[max(PF)], with B a “small” constant: the starting perturbation should typically scale with the distance between eigenvalues in the complex plane, the penalty of a too small B has to be linear (in algorithmic steps); it is the scaling of the “oriented random walk” that we want the eigenvalues to follow toward “the nearest EP”, admittedly a loosely defined concept.

**Figure 6:**
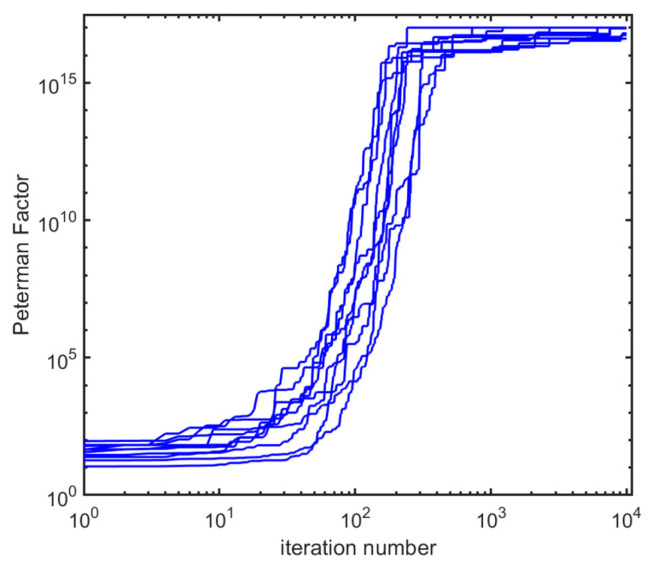
Convergence plot of the algorithm leading to reaching an EP from arbitrary random complex matrices of size N=24. At each iteration, a perturbation is added. If max(PF) is enhanced, the perturbed version is retained; otherwise not. Hence the plateaus when clusters of enhancement failures line up (this may occur in particular after large enhancements, since in this case, it is requested to lower the norm of the perturbation more than achieved automatically to continue the path; the steps are temporarily too large and overshoot the target). The perturbation norm is reduced inversely proportionally to max(PF) to perform a “soft arrival” on the EP. Ten different cases are shown here, with consistent trends, essentially spotting the EP and its huge enhancement after 100 or 200 steps. The ceiling is numerical; it does not carry information above $\max(PF)\sim 10^{17}$.

We show on Figure 6 the result in terms of the max(PF(q)) trajectory vs. iteration number q, on a log–log scale, for a set of 10 starting random matrices. We level the upper part of the plot to $\max(PF)\leq 10^{17}$, which corresponds qualitatively to a numerical accuracy limit whereby there is no more physical information to be gained. All trajectories make large improvements in the range q~10–100, and reach the asymptote around q values of a few hundred, the residual plateauing improvements likely having more to do with numerics.

There are numerous issues that could be further addressed: whether different paths from the same matrix go to the same EP and at the same speed. Such issues have their own interest, but they would be more interesting in a specific context, justifying the quest of EPs in a specific manner. Probably, the “nearest” generic issue, though, would be to wonder about the locus of EP, namely their spectral modulus |z|: is it possible to skew the algorithm to favor the higher |z| EPs, and when does the behavior substantially change from the one exemplified here? We hypothesize that when the modulus approaches the semicircle asymptotic limit ($\left|z\right|\to \sqrt{2}$ for our complex matrices), the quest becomes much more lengthy with the agnostic algorithm used here, based on random perturbation. Perturbations associated with matrices having most of their eigenvalues in a ring close to this limit could be investigated as an example of several possible avenues.

**Figure 7:**
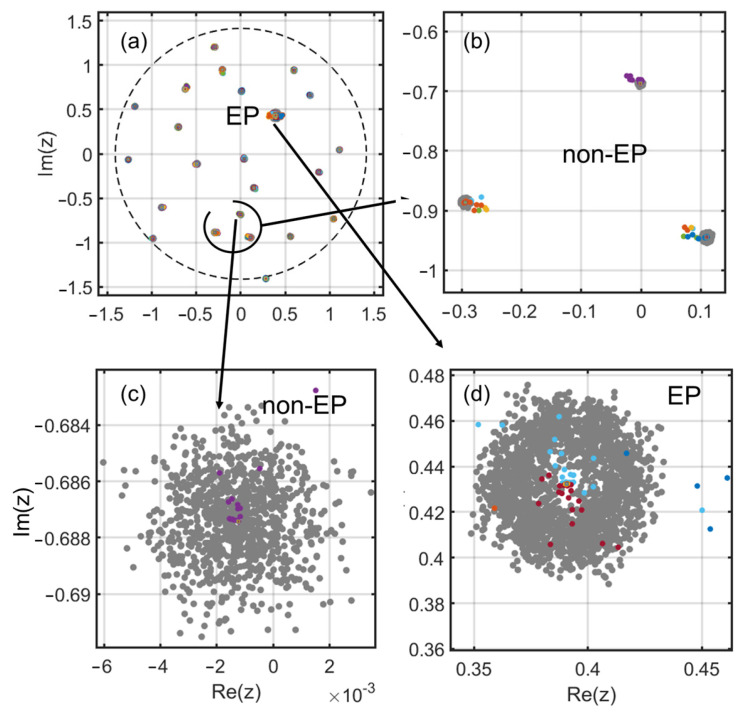
(**a**–**d**) Pseudo-spectrum and assessment of the “EP path” in the complex plane. The final converged matrix, with its EP, is tested against the pseudospectral technique. In (**a**–**d**) the cloud of gray dots is the pseudospectral “result”, while colored dots are associated with the path made from the initial matrix to the final one. (**a**) Complex plane with $\sqrt{2}$-radius circle. Changes are quite modest for most eigenvalues since colored dots and grey dots are clustered at the global scale; it can be said that the initial matrix has been only gently perturbed overall; (**b**,**c**), zooms on non-EP cases, the colored dots lie within typically less than 10 times the pseudospectral radius, giving a more precise basis to the qualitative claim. In the most enlarged zoom (**c**), the iteration points quickly approach the target, albeit not in a straight line, as is logical for a series of “agnostic” perturbations. In (**d**) the attained EP is visualized; the pseudospectral cloud is now much larger and depleted at the center. This is the classical result due to the square-law nature of the perturbation response in the complex plane. The shift from the initial matrix and the pseudo-spectral radius are now of similar order of magnitude. Note that the EP is not at the edge of the circle; it is at less than half the $\sqrt{2}$ spectral radius.

To visualize the result in terms of eigenvalues in the complex plane, we end this section by showing on Figure 7 the superimposition of a typical pseudospectral plot [35] of one of the “converged” matrices of Figure 5, together with sampled points along the trajectory. We use a perturbation of norm ${8\sqrt{2}\;\times 10}^{-4}$ for the pseudospectral plot (black dots show perturbed eigenvalues) and add colored dots corresponding to the eigenvalues along the algorithm path. The general view shows that only one EP has emerged, and the colored dots show that the initial matrix has, overall, only been marginally disturbed. Three zoomed areas show this in more detail. The spread of the pseudospectral eigenvalues with a central hole is typical of the first-order EP being reached.

## 4. Discussion

Our goal was, to put it simply, to understand what it means that an NH operator can be perturbatively modified so as to produce an EP. While EPs in low-dimensional systems are tackled by their occurrence along a parametric path of one of the physical parameters, we have purposely remained “agnostic” about a parametric path. We have only considered how a set of stochastic perturbations could be used to drive the system to a “nearby” EP in the sense that most non-affected eigenvalues only evolve marginally in the complex plane, while those merging in the EP evolve on a broader scale, of the order of the inter-eigenvalue separation in the complex plane.

The main distinction in the above study has been between complex matrices and real (Ginibre) NH matrices. EPs are highly favored on the real axis of the complex plane for the latter case, which calls for a specific algorithm, not “agnostic”, as they would first work from those eigenvalues more concerned with those close to (or inside) the real axis (the central line on the real axis visible in the inset of Figure 1). We did not need any such guessing in the case of complex matrices. The simple algorithm used here has been shown to converge in a reasonable number of steps (pending more improvement efforts; about one hundred to depart, another few hundred to safely reach a numerical EP).

Among the next issues to be tackled are (i) the scaling law of the departure effect; (ii) the capability to apply the same quest to intermediate complex matrices, i.e., those whose imaginary part is weaker than the real part (a physically important subclass) and whose spectrum in the complex plane is an ellipse; and (iii) the capability to run the same kind of algorithm but privileging, for instance, the largest eigenvalues (whose PF tend to be more rigid and much less prone to high values than the “mid-range” eigenvalues).

Finally, one can question the use of a relatively small matrix to provide the numerical example. A N=24 square complex matrix contains over 1150 real parameters (entries), though. In many real-life NH systems, there are rarely more than a few dozen “knobs” to adjust to play with the operator. In acoustics, the examples we have provided typically involve two dozen oscillators. A similar number was chosen by studies on neural networks [3].

But, conversely, if a photonic system has its NH part (gain and loss, as in parity-time symmetry) managed through a common 2D array device, such as a spatial light modulator (SLM), then the number of degrees of freedom increases to $N=10^{6}$. Adaptive optics is another domain whereby wavefronts are reconfigured for maps of such size. The whole system (sensor + actuators) can plausibly be seen as one described by an NH operator.

In all these systems, the expected advantages would be those exemplified for sensing. The system could be perturbatively tuned to exhibit larger sensitivity for targeted input vectors, or targeted spectral domains, to help, for example, extract a first signal from a noisy series and further establish a high-sensitivity communication channel that can be nevertheless later reconfigured.

A last set of possible avenues is to wonder what would be the algorithm that would lead to multiple EPs (triple EP, i.e., EP3), for instance [17,18,19]. One possible strategy is to start from a normal order-2 EP and to track the increase in PFs restricted to all other vectors, with possibly a substantial “nudge” to favor the increase in PF of vectors that tend to merge with those of the EP. An extra metric in the algorithm would be requested, for instance, minimization of the scalar product of the targeted vectors with the (merged) EP vectors (if states are normalized and not the biorthogonal product, cf. the remark in the Introduction).

## 5. Conclusions

We explored the Petermann Factors (PFs) of NH random matrices, in the spirit of producing more easily grasped trends than those in mathematical form in the existing literature. We chose small matrices and large samples rather than the opposite to sample the tails of the obtained distribution on larger ranges. The kind of scaling laws that we have evidenced, while not firmly mathematically justified, should be useful to researchers undertaking exploration of this domain. We have outlined the large differences between complex matrices and real (Ginibre) matrices due to the specific role, in these latter, of the real axis. The role of this axis favors EPs implicitly for real matrices, while it does not for complex ones.

The specific issue of “spawning” EPs from a non-EP or “near-EP” operator has been considered, especially in the complex matrix case. A simple agnostic algorithm performs in a number of steps that seems to scale with the matrix size N, the equivalent of the “healing” process discussed in the previous decade [25,26].

We have schematically discussed variants of the issues tackled in this investigation: “weakly complex” matrices, higher-order EPs, and “nudging” the algorithm to target EPs with sufficiently large spectral radius |z|. A last undiscussed and further avenue would be to consider non-diagonal overlaps [29] rather than diagonal ones considered here, all the more so if some specific contexts would justify extra interest.

## Data Availability

Data and programs are available upon reasonable request.

## Abbreviations

The following abbreviations are used in this manuscript:

EP	Exceptional Point
NH	Non-Hermitian
PF	Petermann Factor

## References

[1] Ashida Y., Gong Z., Ueda M. (2020). Non-Hermitian Physics. Adv. Phys..

[2] El-Ganainy R., Makris K.G., Khajavikhan M., Musslimani Z.H., Rotter S., Christodoulides D.N. (2018). Non-Hermitian Physics and PT Symmetry. Nat. Phys..

[3] Hennequin G., Vogels T.P., Gerstner W. (2014). Optimal Control of Transient Dynamics in Balanced Networks Supports Generation of Complex Movements. Neuron.

[4] Lalanne P., Yan W., Vynck K., Sauvan C., Hugonin J.-P. (2018). Light Interaction with Photonic and Plasmonic Resonances. Laser Photonics Rev..

[5] Benisty H., Greffet J.-J., Lalanne P. (2022). Introduction to Nanophotonics.

[6] Bliokh Y.P., Freilikher V., Shi Z., Genack A.Z., Nori F. (2015). Hidden Modes in Open Disordered Media: Analytical, Numerical, and Experimental Results. New J. Phys..

[7] Eisenbach A., Bliokh Y., Freilkher V., Kaveh M., Berkovits R. (2016). Transmission Resonances Anomaly in One-Dimensional Disordered Quantum Systems. Phys. Rev. B.

[8] Liang Y., Gaimard Q., Klimov V., Uskov A., Benisty H., Ramdane A., Lupu A. (2021). Coupling of Nanoantennas in Loss-Gain Environment for Application in Active Tunable Metasurfaces. Phys. Rev. B.

[9] Liang Y., Bochkova E., Burokur S.N., de Lustrac A., Benisty H., Lupu A. (2024). Engineering of the Fano Resonance Spectral Response with Non-Hermitian Metasurfaces by Navigating between Exceptional Point and Bound States in the Continuum Conditions. Opt. Express.

[10] Dubrovina N., Liang Y., Gaimard Q., Brac de la Perrière V., Merghem K., Benisty H., de Lustrac A., Ramdane A., Lupu A. (2024). Electrically Injected Metamaterial Grating DFB Laser Exploiting an Ultra-High Q Electromagnetic Induced Transparency Resonance for Spectral Selection. Adv. Funct. Mater..

[11] Helmlinger A. (2014). Why Simple? Topology and Spread of the Double Tenor Pan (Trinidad and Tobago). Anthropol. Et Société.

[12] Monteil M., Thomas O., Touzé C. (2015). Identification of Mode Couplings in Nonlinear Vibrations of the Steelpan. Appl. Acoust..

[13] Rüter C.E., Makris K.G., El-Ganainy R., Christodoulides D.N., Segev M., Kip D. (2010). Observation of Parity–Time Symmetry in Optics. Nat. Phys..

[14] Makris K.G., El-Ganainy R., Christodoulides D.N., Musslimani Z.H. (2011). *PT*-Symmetric Periodic Optical Potentials. Int. J. Theor. Phys..

[15] Benisty H., Degiron A., Lupu A., De Lustrac A., Chénais S., Forget S., Besbes M., Barbillon G., Bruyant A., Blaize S. (2011). Implementation of PT Symmetric Devices Using Plasmonics: Principle and Applications. Opt. Express.

[16] Chen W., Kaya Özdemir Ş., Zhao G., Wiersig J., Yang L. (2017). Exceptional Points Enhance Sensing in an Optical Microcavity. Nature.

[17] Wiersig J. (2020). Review of Exceptional Point-Based Sensors. Photon. Res..

[18] Wiersig J. (2023). Petermann Factors and Phase Rigidities near Exceptional Points. Phys. Rev. Res..

[19] Kullig J., Wiersig J., Schomerus H. (2025). Generalized Petermann Factor of Non-Hermitian Systems at Exceptional Points. Phys. Rev. Res..

[20] Schomerus H. (2024). Eigenvalue Sensitivity from Eigenstate Geometry near and beyond Arbitrary-Order Exceptional Points. Phys. Rev. Res..

[21] Takata K., Nozaki K., Kuramochi E., Matsuo S., Takeda K., Fujii T., Kita S., Shinya A., Notomi M. (2021). Observing Exceptional Point Degeneracy of Radiation with Electrically Pumped Photonic Crystal Coupled-Nanocavity Lasers. Optica.

[22] Montag A., Kunst F.K. (2024). Symmetry-Induced Higher-Order Exceptional Points in Two Dimensions. Phys. Rev. Res..

[23] Rahmani A., Opala A., Matuszewski M. (2024). Exceptional Points and Phase Transitions in Non-Hermitian Nonlinear Binary Systems. Phys. Rev. B.

[24] Weis C., Fruchart M., Hanai R., Kawagoe K., Littlewood P.B., Vitelli V. (2025). Generalized Exceptional Points in Nonlinear and Stochastic Dynamics. Phys. Rev. Res..

[25] Benisty H., Yan C., Degiron A., Lupu A. (2012). Healing Near-PT-Symmetric Structures to Restore Their Characteristic Singularities: Analysis and Examples. J. Light. Technol..

[26] Nguyen N.B., Maier S.A., Hong M., Oulton R.F. (2016). Recovering Parity-Time Symmetry in Highly Dispersive Coupled Optical Waveguides. New J. Phys..

[27] Schomerus H., Frahm K.M., Patra M., Beenakker C.W.J. (2000). Quantum Limit of the Laser Line Width in Chaotic Cavities and Statistics of Residues of Scattering Matrix Poles. Phys. A Stat. Mech. Its Appl..

[28] Chalker J.T., Mehlig B. (1998). Eigenvector Statistics in Non-Hermitian Random Matrix Ensembles. Phys. Rev. Lett..

[29] Bourgade P., Dubach G. (2020). The Distribution of Overlaps between Eigenvectors of Ginibre Matrices. Probab. Theory Relat. Fields.

[30] Fyodorov Y.V., Mehlig B. (2002). Statistics of Resonances and Nonorthogonal Eigenfunctions in a Model for Single-Channel Chaotic Scattering. Phys. Rev. E.

[31] Fyodorov Y.V. (2018). On Statistics of Bi-Orthogonal Eigenvectors in Real and Complex Ginibre Ensembles: Combining Partial Schur Decomposition with Supersymmetry. Commun. Math. Phys..

[32] Janik R.A., Nörenberg W., Nowak M.A., Papp G., Zahed I. (1999). Correlations of Eigenvectors for Non-Hermitian Random-Matrix Models. Phys. Rev. E.

[33] Akemann G., Förster Y.-P., Kieburg M. (2020). Universal Eigenvector Correlations in Quaternionic Ginibre Ensembles. J. Phys. A Math. Theor..

[34] Schomerus H. (2017). Random Matrix Approaches to Open Quantum Systems. Stochastic Processes and Random Matrices.

[35] Trefethen L.N., Embree M. (2020). Spectra and Pseudospectra the Behavior of Nonnormal Matrices and Operators.

